# Changes in social interaction, social relatedness, and friendships in Education Outside the Classroom: A social network analysis

**DOI:** 10.3389/fpsyg.2023.1031693

**Published:** 2023-02-02

**Authors:** Jan Ellinger, Filip Mess, Joachim Bachner, Jakob von Au, Christoph Mall

**Affiliations:** ^1^Associate Professorship of Didactics in Sport and Health, TUM Department of Sport and Health Sciences, Technical University of Munich, Munich, Germany; ^2^Institute of Natural Sciences, Geography and Technobiology, Heidelberg University of Education, Heidelberg, Germany

**Keywords:** Education Outside the Classroom, social interaction, social relatedness, natural environments, social network analysis, children

## Abstract

**Introduction:**

Social interaction is associated with many effects on the psychological level of children such as mental health, self-esteem, and executive functions. Education Outside the Classroom (EOtC) describes regular curricular classes/lessons outside the school building, often in natural green and blue environments. Applied as a long-term school concept, EOtC has the potential to enable and promote social interaction. However, empirical studies on this topic have been somewhat scant.

**Methods:**

One class in EOtC (*N*  = 24) and one comparison class (*N* = 26) were examined in this study to explore those effects. *Statistical Actor-Oriented Models* and *Exponential Random Graph Models* were used to investigate whether there are differences between EOtC and comparison class regarding changes over time in social interaction parameters; whether a co-evolution between social interaction during lessons and breaks and attendant social relatedness and friendships exists; whether students of the same gender or place of residence interact particularly often (homophily).

**Results:**

Besides inconsistent changes in social interaction parameters, no co-evolutional associations between social interaction and social relatedness and friendships could be determined, but grouping was evident in EOtC. Both classes showed pronounced gender homophily, which in the case of EOtC class contributes to a fragmentation of the network over time.

**Discussion:**

The observed effects in EOtC could be due to previously observed tendencies of social exclusion as a result of a high degree of freedom of choices. It therefore seems essential that in future studies not only the quality of the study design and instruments should be included in the interpretation – rather, the underlying methodological-didactic concept should also be evaluated in detail. At least in Germany, it seems that there is still potential for developing holistic concepts with regards to EOtC in order to maximize the return on the primarily organizational investment of implementing EOtC in natural environments.

## 1. Introduction

Social interaction and peer relations have been a central topic in social and developmental psychology for years (Hartup, 1999; Hay et al., 2018). From this perspective, the crucial role played by social factors in children’s development should not be underestimated (Seppala et al., 2013). If the social needs of children, such as having a sense of connection and building trust in relations with significant others, remain unsatisfied in the long term, this could lead to the development of mental disorders (Pachucki et al., 2015; McNamara et al., 2017). The importance of such skills is also emphasized with regard to 21st century skills (Chalkiadaki, 2018; Hirsh-Pasek et al., 2020; van Laar et al., 2020). Experiencing social situations that contain the potential to develop social skills in children’s lives, is therefore of tremendous relevance (Hartup, 1989). School life can be seen as a social situation on both a macro (whole school community) and micro (situation in class) level (Sarason and Klaber, 1985; Ma, 2018). Social psychology pursues the task of looking at and analyzing the connections between social situations shaped by social interaction and individual characteristics, behavior or experiences (Baron et al., 2000). In this context, we understand social interactions to be an active process between individuals on an interpersonal level (Simmel, 2013). From this, according to Ryan and Deci (2017), the passive state of social relatedness (as one of the basic psychological needs assumed to be central for the individual), which alludes to a feeling of belonging, can result. Following this, social relatedness also expresses the perceived connection to other people and, thus, inclusion in a particular group. With that being largely based on a particular individual and non-standardized feeling, other aspects like the number of friends cover solely quantitative impressions of embeddedness in a certain group (Fullerton and Ursano, 1994). Both can be the result of previous social interaction as the core of a social situation, such as school life (Baron et al., 2000).

### 1.1. Research on social interaction in children

Social interaction is important to various aspects of children’s life, such as well-being (Lee et al., 2020) and mental health (Li et al., 2020), self-esteem (Harris and Orth, 2020), learning outcomes (Hurst et al., 2013), and executive functions (Moriguchi, 2014). The school setting provides an extremely valuable platform for observing, analyzing, and affecting social interaction since it represents the most important place for social interaction among young people (McNamara et al., 2017; García-Carrión et al., 2019). Children and adolescents spend a great deal of time here. Additionally, the majority of children can be reached and have the opportunity to participate in certain programs regardless of their background. Furthermore, childhood seems to be particularly suitable for forming social bonds between peers (Gifford-Smith and Brownell, 2003). However, there continues to be a paucity of intervention studies that address all students equally, with the explicit goal of improving social interaction between the students with possible effects on social relatedness and friendships.

In childhood, one’s own gender and the gender of peers in particular prove to be significant for socialization and friendships (Maccoby, 1988; Block and Grund, 2014). Confrontation with members of the same gender fulfills various important functions in the development of one’s own (gender) identity [as does later confrontation with the other biological gender (depending on sexual orientation); Ridgeway and Smith-Lovin, 1999; Powlishta, 2004; Perry et al., 2019]. During late childhood and early adolescence, a strong tendency for girls to interact more with girls and for boys to interact more with boys is evident (Maccoby, 1988; Shrum et al., 1988). Apart from these tendencies, which are typical in the course of development, the school setting also provides additional opportunities to influence social interaction between students. This could include the creation of special meeting and social spaces in school, or the implementation of specific social activities. Also, entire teaching concepts could be used in order to influence social interaction. One of those is “Education Outside the Classroom” (EOtC).

### 1.2. Social interaction in Education Outside the Classroom

EOtC is a teaching concept that is – in line with the curriculum – regularly carried out in a natural or cultural environment. EOtC is often practiced for a duration of several consecutive months and up to whole years or more. In most studies, explicitly investigating EOtC, this concept has to be applied at least bi-weekly for four or more lessons. If EOtC is sustainably established at a school, it can enrich the daily school routine as an applicable long-term concept with manageable extra costs (Bentsen et al., 2009, 2021). EOtC lessons are said to be experimental, student-centered, and there is an increased use of group and partner work (Bentsen et al., 2021; Ellinger et al., 2022b). They lead to higher levels of physical activity (Schneller et al., 2017), especially if they are conducted in natural environments (Bølling et al., 2021), which is in part associated with a healthy pattern of cortisol secretion that indicates less stress over the course of the day (Becker et al., 2019). In addition, it can be assumed, that EOtC promotes intrinsic school motivation (Bølling et al., 2018; Ellinger et al., 2022a) and well-being of the students (Jørring et al., 2019).

Moreover, EOtC offers numerous ways of affecting students’ social interaction. Even with regard to natural spaces in themselves, it is worthwhile to consider facets of social interaction from different perspectives (Waite et al., 2016; Torkos, 2017; Roberts et al., 2020). Independently of school lessons, it has occasionally been demonstrated that children’s intensive engagement in and with nature in groups (e.g., free outdoor play, adventure therapy, seated relaxation, and orienteering) can improve several social aspects such as mutual trust, social cohesion, social functioning, relationship to peers, and cooperation (Doucette, 2004; Roe and Aspinall, 2011; McArdle et al., 2013; Mygind et al., 2019). The availability of natural spaces in the immediate living environment also seems to be able to influence social factors and consequently health (Faber Taylor et al., 1998; Sugiyama et al., 2008). These different and partly specific outcomes limit the comparability, but in sum, there is a clear tendency that both adults, but also children can benefit from group activities in and with nature with regard to their social skills (Mygind et al., 2019; Fyfe-Johnson et al., 2021). As EOtC often takes place in natural environments, these possible benefits of natural environments are combined with a special teaching situation, enabling substantial restructuring, simplification, and promoting cooperative group work, because rigid structures of traditional school teaching such as a fixed seating and desk arrangement are eliminated. Therefore, EOtC offers specific conditions that may help to improve social relations (e.g., play, interaction, student-centered tasks, and cooperation; Hartmeyer and Mygind, 2016; Glackin, 2018). This produces the result that the lessons often differ significantly from traditional indoor school lessons. Regarding social aspects, previous research has shown that EOtC could lead to more positive peer relations than traditional teaching concepts (Mygind, 2009) and to the development of new positive peer affiliations (Bølling et al., 2019), which could be explained by the character of EOtC (Fägerstam, 2014): In the indoor setting of the classroom, social contacts are often limited to the students sitting near to each other. Since the usual spatial forms of organization are largely absent in EOtC, it can be assumed that other ways of teaching and new social constellations are facilitated, as already shown in surveys regarding social forms in EOtC (Ellinger et al., 2022b), as well as opportunities for informal interaction are created (Jørring et al., 2019). This could lead to intensified movement in space and its appropriation by the students, especially in group constellations – while teaching the same subject matter as during traditional indoor teaching (Mall et al., 2021). Additionally, it has been shown that curriculum-compliant teaching in natural settings can lead to comparatively increased (facets of) social relatedness (Dettweiler et al., 2017), which could be a result of those new or intensified opportunities for social interaction. Undoubtedly, however, there is still a great demand for further studies on this promising topic (Becker et al., 2017).

The central assumption of this study is that EOtC, due to its integration into natural environments, as well as the utterly changed teaching situation, enables not only the implementation of new forms of social interaction, but also more social interaction. This could be captured by the numerical number of classmates with whom a particular child interacts in the context of school or class. Change in social interaction in turn could have an impact on the experience of social relatedness and friendships. In the present study, we therefore aim to map social relatedness and the structure of friendships in addition to assessing social interaction in the class, which represents a novelty in EOtC. We pursue the approach of viewing these social constructs not as factors or mediators for other behaviors but as the main outcomes, which in this context is still rare. In summary, we hypothesize that the introduction of EOtC in natural environments could lead to intensification and restructuring of the network of social interaction in a school class, which is the main focus in this article. With that, also changes over time in social relatedness, as well as friendships seem reasonable. Furthermore, it seems possible that, due to the preference of same-gender interactions in children and adolescents (Maccoby, 1988; Shrum et al., 1988), gender can also influence their social interaction. This, in turn, is of scientific interest given the relevance of engagement with other genders for child development (Ridgeway and Smith-Lovin, 1999; Perry et al., 2019). Additionally, place of residence could also be relevant. This assumption is partly due to the special situation regarding many secondary schools in Germany: When children move from primary to secondary school, this often involves a change of location, as secondary schools are often located only in (larger) cities, so that children from rural areas have to be transported here. This means that children from different cities and villages come together in secondary school. We assume that children who, for example, move from the same village to the same secondary school at the same time have a closer connection because they share their way to school or they may have already been to elementary school together.

This study aims to investigate the following central hypothesis: (a) The students within a EOtC class show increases in social interaction over time, which go beyond those changes in a comparison class without EOtC. Additionally, this study explores the interrelations of those changes with other social factors. Therefore, two sub-hypotheses are set up: (b) There is a co-evolution of the changes of social interaction and social relatedness and friendships over time in both classes; (c) Gender and place of residence partly explain the positions of actors in the network in both classes.

## 2. Materials and methods

### 2.1. Intervention

In this study, a class with EOtC is compared to a class without EOtC. In the following, it is described how EOtC was implemented at the cooperating school and how the environment the EOtC took place at can be characterized. Please see also Table 1 for a detailed schedule of an exemplary EOtC-day.

**Table 1:** Schedule of an exemplary EOtC-day.

Schedule	Content	Environment
7.55–8.15 am	EOtC class and two teachers meet at school; check for attendance; important announcements	School building or grounds
8.15–8.30 am	Walking from school to the natural environments nearby the city	Residential area, agri-cultural land and meadows
8.30–8.35 am	Arrival at forest; class splitting up (half of the class with one teacher each); arrangement of a meeting point	Natural environments like forest, clearings and meadows
8.35–8.45 am	Groups walking to the specific places of teaching; preparation of the teaching materials	
8.45–10.15 am	Teaching in the subject of German or Biology; shorter breaks	
10.15–10.45 am	Walking back to the meeting point; longer break	
10.45–10.55 am	Walking to the specific places of teaching; preparation of the teaching materials	
10.55 am–12.25 pm	Teaching in the subject of German or Biology; shorter breaks	
12.25–12.35 pm	Walking back to the meeting point; check for attendance	Natural environments like forest, clearings and meadows
12.35–12.50 pm	Walking back to school together (whole class with both teachers)	Residential area, agri-cultural land and meadows
Approx. 12.55 pm	Official closing of the school day	School grounds

Although the forest used for the EOtC is accessible from the city and the school within approximately 15–20 min walking distance, it can clearly be classified as a natural space or natural environment following consolidated definitions within the field of outdoor play, learn, and teach (Lee et al., 2022). On the fixed EOtC day during our study, the students and two teachers first met in the school building. There, attendance was checked and, if necessary, important instructions for the day were given. Then, the class set off on foot into the forest. Often the students talked among themselves or with the teachers or played little games on the way there. However, there were no organized activities along the way. After a few meters in the residential area, the path the class used was characterized by agricultural land and open meadows. Once in the forest, the class was divided to be taught initially for two school hours (90 min) in one subject and, after a change of groups, in a second subject (90 min) by the two accompanying teachers. In the case of this investigation, the subjects were German and Biology. The learning site visited in the case of this study is a forest with old trees and unstructured vegetation that, apart from the usual forest management, is left in its natural state. The lessons did not take place in a clearly defined outdoor classroom. Accordingly, there were no benches or a blackboard. The lessons were realized in different places where the children sat or stood on tree trunks or on the ground and it was the responsibility of the respective teacher how he or she organizes the lesson – e.g., whether movement breaks were specifically built in or special social forms were used. After those two different lessons, the group walked back to the school building together, where the school day ends.

### 2.2. Participants and procedure

Two fifth-grade classes of a German public school participated in this study (*N* = 50). The school cooperating in this study has one fifth-grade class that received at least 4 h of curriculum-compliant schooling in the nearby forest [EOtC class; *n* = 24 (female = 45.8%)] for the whole school year at one fixed day of the week (first year of conducting EOtC at that school). The comparison class [*n* = 26 (female = 46.2%)] received only indoor teaching. Students of the EOtC class came from five different places of residence in and around the city where the school is located. In the comparison class, there were six different places of residence. Since the class assignment depends on the registration of the parents of their children in the respective class, no randomization could be carried out. We conducted our surveys at this school at two time points during the 2019/2020 school year: One month after the beginning of the school year (to wait for a certain period of acclimatization in the new class at the beginning) (T1) and after 5 months (T2). Due to the pandemic-related school closures, the planned third time point at the end of the school year could not take place. This article is part of a larger research project.^1^ First results concerning school motivation, health-related quality of life, and the satisfaction of basic psychological needs are published elsewhere (Ellinger et al., 2022a). The differences in the sample size of the two studies result from different methods applied in both studies. The partial secondary analysis of basic psychological needs (social relatedness) is based on the different research questions of the articles.

### 2.3. Instrumentation

#### 2.3.1. Social interaction

We developed our own questionnaire to collect data on social networks, following common practices (Borgatti et al., 2018). The questionnaire contained two different initial questions and a class list for each question. The students were asked to tick all the students on the lists to whom the initial questions applied. The questions asked were*: (a) With whom do you regularly learn and work during the lessons? (b) With whom do you regularly play during the school breaks?* For the EOtC class, the questions were phrased to refer explicitly to EOtC, the comparison class answered the questions in terms of normal indoor lessons. We understand these questions to represent social interaction and act as the basis for our network data (network questions).

#### 2.3.2. Social relatedness

We used a German translation of the *Basic Psychological Needs Scale* (*BPNS*; La Guardia et al., 2000; Hanfstingl et al., 2010) to assess social relatedness using a 5-point Likert scale (e.g., *I have a good relationship with my classmates*). Social relatedness (Cronbach’s *α* = 0.77) is one of the subscales of the *BPNS*; the results of the other subscales were not of interest for the present analysis and were therefore not included in the analysis. The factorial validity of the *BPNS* and its subscales has been frequently addressed in the past and is in general well-supported both theoretically and statistically [CFI (comparative fit index), TLI (Tucker-Lewis index) > 0.95; Deci and Ryan, 2000; Wang et al., 2019]. Following the recommendations by the authors of the statistical method used (*cf.* the following chapter *Statistical data analysis*), the raw values of social relatedness were divided into seven even categories (Nynke et al., 2019).

#### 2.3.3. Friendships

We also asked the students which of their classmates they would describe as a friend. With this information, we calculated the total number of friends (friendships) and the proportion of mutually declared friendships (mutual friendships). We divided those into three categories (1 = one is nominated as a friend by more than 20% more classmates than the other way around [meaning, the corresponding person is perceived as a friend by more classmates, as he or she would refer to as a friend], 2 = one is nominated as a friend by about as many [+/− 20%] classmates as the other way around, 3 = one is nominated as a friend by more than 20% less classmates as the other way around). With that, we managed to receive three groups about the same size. This categorization is not intended to indicate a qualitative classification but to facilitate the interpretation of the results.

We consider, that social relatedness and (mutual) friendships complement each other to capture an overall impression of perceived embeddedness in the social structure of the class: One representing more the feeling of inclusion, independent of the number of personal contacts (social relatedness) and one the quantitative measurement of the number of a certain type of social reference persons (friendships). In addition, we assessed the age, gender, and place of residence of the participants in a demographic questionnaire.

### 2.4. Statistical data analysis

In our analysis, the relationship structures generated from the answers to the network questions represent the networks themselves. This means that if person A has indicated that he or she works together with person B during lessons, there is a connection (edge) between person A and B (nodes) in the logic of a network. Thus, a network for the whole class results from the totality of the answers of all students. We treat social relatedness and (mutual) friendships as dynamic attributive variables of the students while defining gender and place of residence as static attributes and covariates. We set up four models, two models each for the EOtC and comparison class representing the two network questions regarding interaction during lessons and breaks.

There are currently two different main approaches for the purpose of statistical network analysis. “Random Exponential Graph Models” (ERGMs) are well-suited to test for the randomness of realized connections in a cross-sectional network analysis (Shumate and Palazzolo, 2010). This means that ERGMs check whether connections in the overall network occur particularly frequently between persons who have the same characteristic of interest (e.g., gender). “Stochastic Actor-Oriented Models” (SAOMs) are feasible for longitudinal network data combined with additional dynamic variables (Ripley et al., 2012). SAOMs therefore combine ERGMs with a temporal component that can also determine randomness (or over-randomness) of connections over a certain period of time. Compared to ERGMs with temporal extension, SAOMs have certain advantages and disadvantages, the relevance of which depends on the type of data collected (Block et al., 2019).

For our analysis of hypotheses (a) and (b), we used the R-package *RSiena* for constructing SAOMs. As key-figures in order to address hypothesis (a), we calculated density, diameter, clustering coefficients, and similarity index. Those are central metrics for gaining an impression of the overall constitution of a network. Since the two classes are almost identical in size and also largely homogeneous in terms of age and gender composition, these parameters can subsequently also be used to compare the results of the EOtC class with the comparison class. We assume that the inclusion of different parameters allows a deeper understanding of the network structures in comparison if only a single metric would be included. To address hypothesis (b), co-evolutional associations (degree and dense triads) were tested. We checked whether the number of the realized edges of a person (degree) correlates with the calculated value of social relatedness and the number of friendships over time. Please see Table 2 or respective basic literature (e.g., Luke, 2015) to clarify these key terms in interpreting social networks. In network analysis, hypotheses like hypothesis (c) are referred to as “homophily” hypotheses, as they test whether nodes that share the same characteristics in relevant aspects (in our case: characteristics of gender and/or place of residence) tend to interact more closely or more often. To address hypothesis (c), we constructed ERGMs using the R-package *ergm* (Hunter et al., 2008). We conducted separate analyses for T1 and T2.

**Table 2:** Central terms in the interpretation of a social network and their meaning.

Term	Meaning
Degree	The degree indicates how many connections a node/actor in total has to others in the network. In most networks, a higher degree is considered better because it increases integration in the network. This has to be assessed differently if the recorded interaction is classified as negative (e.g., bullying). Whether, in our case, higher degree is also associated with higher social relatedness (and thus would be considered clearly positive) is to be tested in our study.
Density	The density of a network indicates the proportion of how many of the maximum possible connections are realized between the nodes/actors. As a result, possible values range from 0 to 1. These are not standardized values, so a difference of 0.2 should be interpreted differently in a particularly dense network (e.g., insults between siblings) than in less dense networks (e.g., insults between work colleagues). Similar to the degree, a higher density is desired in most cases and can, therefore, be classified as better. In our case, the classification as “good” for a higher density depends, analogously to degree, on its associations with further parameters.
Diameter	For a network, it is important how many intermediate steps (other nodes/actors) connect each node/actor to every other node/actor, whereby a small number is considered as better. In order to determine the diameter of a network, a two-step procedure is followed. For every actor, the minimal distances (*via* the existing edges) to reach each of the other actors are identified. The longest of these minimal distances then represents the diameter. Since this is about absolute and not relative numbers, a comparison only makes sense if the networks to be compared have (roughly) the same number of nodes/actors. In our case, for example, a lower diameter could imply that lesson-relevant information reaches all students more quickly.
Clustering coefficient, transitivity, and dense triad	The calculation of clustering coefficient, transitivity, and dense triads provides information (probability between 0 and 1) about whether the nodes/actors in a network tend to form groups (meaning: A being connected with B and C also implies a high probability of B being connected with C). High values indicate that there is increased clique or triad formation. Depending on the background of the network, this can be assessed as positive or negative. As the children in the EOtC are able to interact more freely with each other, it seems possible that this will either lead to the strengthening of existing groups or to a break-up of them. It is important to note that the calculation of the clustering coefficient (based on clustering for each node) on the one hand and transitivity and dense triads (proportion of triangles compared to total number of connected triples), despite their similarity, can show different values and even different tendencies.
Similarity index	Corresponding values provide information about how similar two networks are. The possible values range from 0 (no similarity) to 1 (identical networks). This could mean two networks of different groups or, like in our case, the same network at two different time points. In that case, one could also talk about a “stability index.” While the density only allows a statement to be made about the proportion of realized connections, the similarity index also considers whether the same nodes/actors remain directly connected over time. Thus, it is possible to have an unchanged density and low similarity at the same time. Therefore, similarity index and density represent important complementary indicators.

## 3. Results

In terms of density, a strong decrease in social interaction during lessons from T1 (0.26) to T2 (0.22) can be observed in the EOtC class (*cf.* Table 3), which runs contrary to hypothesis (a) and the trends in the comparison class (T1: 0.2; T2: 0.22). However, a strong increase in density is observed concerning social interaction during breaks from T1 (0.3) to T2 (0.36) in the EOtC class, whose relevance is also underlined by statistical significant results of the SAOM analysis regarding associations over time [*cf.* Table 4 (*β* = −1.02; *SE* = 0.53; *p* < 0.05)]. The values in the comparison class regarding social interaction during breaks drop slightly (T1: 0.33; T2: 0.31). Measured by the Jaccard similarity index (Snijders et al., 2010), both groups show moderate stability over time in the respective forms of interaction. There is an apparent network fragmentation in social interaction both during lessons and during breaks, which complicates the interpretation of the diameter and the clustering coefficients (Luke, 2015). Please see the up-coming section regarding gender homophily for more information on those fragmentations.

**Table 3:** Network parameters.

Education Outside the Classroom					Comparison				
	Density	DM	CC	JSI		Density	DM	CC	JSI
Model 1 (Lessons)					Model 3 (Lessons)				
T1	0.26	6	0.67			0.20	5	0.47	
T2	0.22	X/3*	0.56			0.22	5	0.41	
				0.467					0.448
Model 2 (Breaks)					Model 4 (Breaks)				
T1	0.30	X/3*	0.74			0.33	4	0.64	
T2	0.36	X/3*	0.84			0.31	4	0.68	
				0.655					0.522

Density (Density), diameter (DM), clustering coefficient (CC) and Jaccard similarity index (JSI) for both groups (Education Outside the Classroom, Comparison) at different time points (T1, T2); Models 1 and 3 concern the interaction during the lesson, models 2 and 4 the interaction during the breaks in the corresponding group.

* In the case of fragmentation of the corresponding network (X), the diameter of the largest fragment was calculated instead.

**Table 4:** Results of testing for structural effects and co-evolutional associations over time.

Education Outside the Classroom					Comparison				
		*β*	SE	*p*			*β*	SE	*p*
Model 1 (Lessons)					Model 3 (Lessons)				
	Transitivity	0.27	0.13	*		Transitivity	0.34	0.26	–
	Density	−1.02	0.53	–		Density	1.06	0.62	–
SR	Degree	0.34	0.70	–	SR	Degree	−0.33	1.19	–
	Dense Triads	−0.12	0.18	–		Dense Triads	0.19	0.80	–
Friends	Degree	0.02	0.21	–	Friends	Degree	0.01	0.44	–
	Dense Triads	0.01	0.05	–		Dense Triads	−0.01	0.21	–
MFC	Degree	−0.11	0.40	–	MFC	Degree	−0.35	3.76	–
	Dense Triads	0.06	0.20	–		Dense Triads	0.49	2.78	–
Model 2 (Breaks)					Model 4 (Breaks)				
	Transitivity	0.54	0.23	*		Transitivity	0.38	0.58	–
	Density	1.88	0.43	*		Density	−1.68	1.59	–
SR	Degree	0.53	0.51	–	SR	Degree	0.50	4.05	–
	Dense Triads	−0.11	0.08	–		Dense Triads	−0.14	0.89	–
Friends	Degree	0.53	1.13	–	Friends	Degree	0.04	0.74	–
	Dense Triads	−0.09	0.23	–		Dense Triads	−0.01	0.33	–
MFC	Degree	0.61	3.96	–	MFC	Degree	0.74	5.33	–
	Dense Triads	−0.16	0.91	–		Dense Triads	−0.06	1.95	–

SR, social relatedness; Friends, friendships; MFC, mutual friends category for both groups (Education Outside the Classroom, Comparison); β = Estimates; SE = Standard error; p = Significance at the corresponding level (* < 0.05); Models 1 and 3 concern the interaction during the lesson, models 2 and 4 the interaction during the breaks in the corresponding group.

In calculating the models concerning hypothesis (b), excellent t-ratios as an indicator for convergence of the models were found in 72.2% of the calculated effects, 27.8% have to be described as reasonable based on established recommendations with no specific pattern occurring (Ripley et al., 2012). In our analysis, we found initial evidence that in EOtC, measured by transitivity, there is a structural tendency to form groups concerning social interaction during lessons (*β* = 0.27; *SE* = 0.13; *p* < 0.05) and breaks (*β* = 0.54; *SE* = 0.23; *p* < 0.05) over time, as also illustrated by the results in Table 4. Given the other results presented, our analysis cannot confirm the existence of co-evolutional associations between the two forms of social interaction on the one hand and social relatedness and (mutual) friendships on the other. Our results, as shown in Table 4, suggest that models 3 and 4 (comparison class) show higher standard errors than models 1 and 2 (EOtC class).

**Figure 1:**
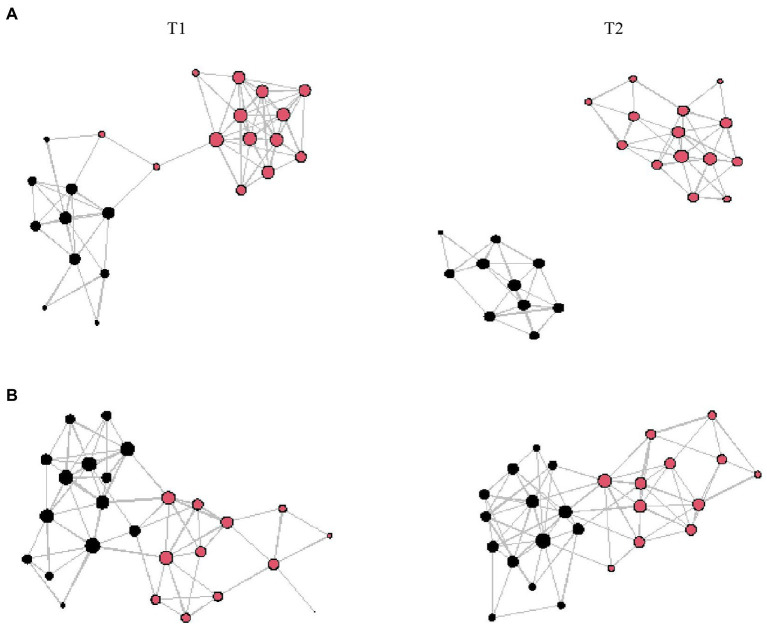
Visualized networks taking gender into account (red represents female students, black represents male students); the size of the dots reflects the degree of connections; the length of the lines does not communicative substantive information; **(A)** Interaction during lessons in EOtC class, **(B)** Interaction during lessons in comparison class; T1 = first time point; T2 = second time point.

**Figure 2:**
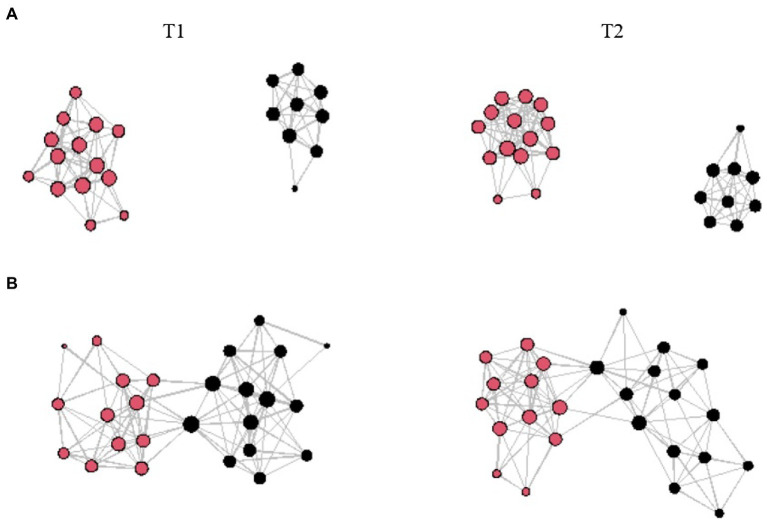
Visualized networks taking gender into account (red represents female students, black represents male students); the size of the dots reflects the degree of connections; the length of the lines does not communicative substantive information; **(A)** Interaction during breaks in EOtC class, **(B)** Interaction during breaks in comparison class; T1 = first time point; T2 = second time point.

Looking at gender homophily regarding hypothesis (c), strong associations for social interaction during the lessons at T1 within students of the same gender are apparent for EOtC class (*β* = 3.85; *SE* = 0.43; *p* < 0.001). For comparison group, that is evident for both during-lesson and during-break social interaction and both time points (Lessons T1: *β* = 2.82; *SE* = 0.32; *p* < 0.001; Lessons T2: *β* = 2.81; *SE* = 0.3; *p* < 0.001; Breaks T1: *β* = 3.50; *SE* = 0.28; *p* < 0.001; Breaks T2: *β* = 3.53; *SE* = 0.29; *p* < 0.001). This is also illustrated in Figures 1 (social interaction during lessons), 2 (social interaction during breaks). Please also see Table 5 for the full results. The EOtC class shows several times that a calculation of gender homophily is not possible. This is because the corresponding networks fragment, and none of these fragments show more than one characteristic – in this case, gender (*cf.* Figures 1, 2). This means that the formerly connected network of the whole class has become two separate networks – one consisting only of boys, one only of girls. In substance, this is to be evaluated as evidencing even stronger homophily than could be expressed by statistical significance. This also applies to the results regarding hypothesis (a) presented above. In the case of residence, significant associations emerge only in the comparison class concerning social interaction during lessons at T2 (*β* = 0.68; *SE* = 0.25; *p* < 0.01).

**Table 5:** Results of testing for homophily effects.

Education Outside the Classroom					Comparison				
		***β***	**SE**	***p***			***β***	**SE**	***p***
Model 1 (Lessons)					Model 3 (Lessons)				
T1	Gender	3.85	0.43	***		Gender	2.82	0.32	***
	Residence	0.26	0.25	–		Residence	0.27	0.28	–
T2	Gender	X	X	X		Gender	2.81	0.3	***
	Residence	−55.8	−2.77	–		Residence	0.68	0.25	**
Model 2 (Breaks)					Model 4 (Breaks)				
T1	Gender	X	X	X		Gender	3.50	0.28	***
	Residence	0.12	0.24	–		Residence	−0.32	0.26	–
T2	Gender	X	X	X		Gender	3.53	0.29	***
	Residence	−0.15	0.24	–		Residence	−0.09	0.26	–

Gender and place of residence (Residence) for both groups (Education Outside the Classroom, Comparison) at different time points (T1, T2); β, estimates; SE, standard error; p = Significance at the corresponding levels (Codes: *** < 0.001; ** < 0.01; * < 0.05); Models 1 and 3 concern the interaction during the lesson, models 2 and 4 the interaction during the breaks in the corresponding group; X = Not possible to calculate a value for the class as a whole because of fragmentation of the corresponding network.

## 4. Discussion

Without a doubt, EOtC must be seen as an investment and not a matter of course in the context of traditional public schools in Germany. This concerns only secondarily financial aspects, but primarily an organizational investment. In sum, the school and the teachers involved have to invest time (e.g., adapting the lessons to the new learning location and travel times) and teaching load (often two teachers for one class) in order to implement the concept. The possible outcomes - or return-on-investments – are, in the best case, positive changes in students and/or teachers. These could relate to learning, health, environmental behavior and much more (Becker et al., 2017). Whether the return on organizational investment is sufficient to justify the concept is at the discretion of the school and the actual effort involved in implementing it. As classroom processes as well as social interaction were only rarely focused on in research on EOtC, we contribute to a deeper understanding of those with the results of our social network analysis. We will discuss those results and how to increase the return-on-investments in EOtC, as well as point out future research implications based on learnings from this study.

### 4.1. Changes in social interaction

Since we observed a decrease in density regarding social interaction during lessons over time in EOtC, but an increase in social interaction during breaks and exactly the opposite tendencies in the comparison class, no clear pattern in favor of the EOtC class can be derived from the present models. The observed trends in the EOtC lessons are surprising considering the existing literature on the influence of natural environments in general and EOtC in particular on social facets (Bølling et al., 2019; Putra et al., 2020). The observed increase in density in social interaction during the breaks in EOtC initially underlines opinions that are based on the assumption that free play in natural spaces positively influences children’s cooperation (Tremblay et al., 2015). Based on the values of the clustering coefficients (*cf.* Table 3) and longitudinal transitivity results (*cf.* Table 4), there appears to be a substantial degree of grouping in the EOtC class. This is in line with previous impressions of social exclusion in EOtC in qualitative studies (Jørring et al., 2019). Previous studies also indicated that existing or missing didactical concepts or guidelines should be taken into account when interpreting results with regard to EOtC in any case (Mygind, 2009; Hartmeyer and Mygind, 2016). Following this, in this case, processes could even occur that favor segregation: EOtC is at least in Germany a relatively new concept, and often initiated as an “educational experiment.” This often leaves teachers with some uncertainties (Barfod, 2018), which could also apply to this case as the studied school year was the first-ever with EOtC being implemented at our cooperation school. Accordingly, the focus is often on the successful delivery of the lesson and less on the targeted mixing of the class. This in turn could lead to teachers leaving the children to organize themselves into groups, which could result in certain group structures being strengthened and established even more, rather than promoting the formation of new interactions. In this respect, a comparison can certainly be drawn with the situation of young teachers in the transition from the “Referendariat” (final practical part of teacher training in Germany) to actual teaching work in the school. Here, too, it can be observed that disruptions (which do occur frequently in reality) of the ideal-typical lesson lead to a focus on the superordinate organization of the school lesson and that desirable didactic requirements (e.g., the promotion of social exchange) are pushed into the background. Consequently, the implementation of these would only be sought again when a certain level of self-confidence in teaching is perceived by the teacher (Miethling, 1986). Teachers may face a similarly new situation when they take the step of implementing EOtC for the first time. Therefore, it remains to be noted that even in a teaching concept that, like EOtC, makes it increasingly easy to use cooperative forms of learning, the integration of possible outsiders does not happen naturally. This conclusion is also in line with other empirical findings and theoretical considerations regarding integration and inclusion in schools (Koster et al., 2009).

In summary, it can be stated that despite the (partly) high organizational investments and the favorable framework conditions in a natural environment, it is not given that expected effects will actually occur. With regard to increases in social interaction, we consider nature-based learning to be a great opportunity, but we also point out the necessity that concepts such as EOtC are not “self-propelling,” but also require concrete didactic-pedagogical principles and guidelines.

### 4.2. Interrelations with other social factors

In this study, we considered the relationships between social interaction and two social facets that have manifold relevance at the psychosocial level but differ in their substantive meaning: Social relatedness as a highly individual and non-standardized feeling of inclusion in the class on the one hand and the number of (mutual) friendships as a purely quantitative representation of social contacts (of outstanding importance) on the other hand. Contrary to hypothesis (b), there are no indications based on our analysis for a substantial co-evolution between the network parameters of social interaction and social relatedness and (mutual) friendships. Thus, the analysis of our sample does not immediately imply that there is a direct association between social interaction and, for example, perceived social relatedness on an individual level over time. Those results apply for both groups. The first network question particularly targeted classroom interaction during the lessons, which can be understood as social interaction. However, social interaction during lessons may be much more related to learning or academic effects than to psychosocial effects such as social relatedness. Suppose one were to understand this question more as an inquiry into cooperative learning in the classroom. In that case, a variety of possible effects on facets of school motivation, cognitive activation, and academic comprehension in students could be identified from empirical research (Gillies and Ashman, 2000; Hänze and Berger, 2007; Fernandez-Rio et al., 2017), but only very rarely on social facets such as bullying (Van Ryzin and Roseth, 2018). Nevertheless, based on previous research we assumed that those cooperative forms of learning might affect social factors (Roseth et al., 2008). In the future, it should be carefully considered which processes the asked questions explicitly address and with which other parameters an association seems possible. However, this explanatory approach does not apply to the social interaction during breaks, as we suspected playing with peers to be fundamentally relevant to social relatedness - a field of research that still offers great potential for future studies. Unfortunately, we do not have any information on how the breaks were organized in the EOtC and the comparison class, which makes further explanations very difficult. Such a classification and description of the social situation should be enabled and considered in future studies.

Concerning hypothesis (c), the results indicate strong gender homophily in both groups for both forms of social interaction, which in the case of the EOtC class even seems to be causal for a fragmentation of the network (*cf.* Figures 1, 2). In general, these results are in line with earlier findings suggesting the importance of having the same gender for the initiation of friendship relationships in childhood and adolescence (Shrum et al., 1988; Block and Grund, 2014). Similar results of the comparison class point to distinctive homophily concerning gender in both forms of social interaction. However, in contrast to the EOtC class, this does not lead to network fragmentation. On the contrary: An increase in the density of social interaction in the comparison class can be observed during lessons, even if corresponding intensifications could not be proven for social interaction during the break. One possible reason for this could be the character of the comparison class. It is for this class to get 45 more minutes of physical education (PE) per week than the other classes, as this class is part of a special school program (PE-profile). This additional lesson of PE was conducted co-educationally, meaning mixed between genders, in contrast to the regular PE lessons. Earlier, the mixing of genders in PE was discussed as being worthwhile (Hills and Croston, 2012), even though a solid scientific basis on the influence of a co-educational approach on social interaction is still lacking. However, it is also explicitly anchored in the curriculum and pedagogical paradigm in the region of our cooperation school that co-educational PE should be utilized to promote gender interaction (Ministerium für Kultus, Jugend und Sport Baden-Württemberg [Ministry of Education, Youth and Sports Baden-Württemberg], 2016). The observed tendency for the genders to be more closely intertwined in the comparison class may be due to this pedagogical paradigm. An equivalent claim of gender interaction does not yet exist for EOtC. Instead, a similar mechanism could come into play here as it has been cited for the changes in social interaction before. The teachers’ focus at our cooperation school might have been primarily on the successful delivery of the lessons. This may have led to students organizing themselves in the context of group work, for example. This, in turn, may have consolidated already existing groups, as reflected in the results of this study as well as a study by Jørring et al. (2019).

It also seems conceivable that the observed effects of homophily with respect to place of residence in the comparison class are due to their affinity for sports: Often, students in PE-profile classes have a relevant sports biography – e.g., many years of membership in sports clubs – as they or their parents actively opt for that school profile. Given the described considerations regarding a closer relationship of the students who come from the same village and possibly already interacted with each other in sports clubs there, these effects could be due to this and apply more to the students within the comparison class.

### 4.3. Limitations and future research implications

We examined EOtC in natural environments as a feasible long-term concept in terms of different facets of social interaction as outcomes. With that, new insights could be gained for research in this area. Subsequent investigations should be aware of and address the limitations and learnings derived from this study. In this study we compared the results of the EOtC class with those of a comparison group that has special characteristics (PE-profile), which offers additional approaches for the interpretation of the results. In the future nevertheless, it would be worthwhile to proceed in a similar way with a real non-treatment group. Second, the sample size must be considered small, which could be the reason for the variance in the level of standard error when comparing the models. However, in this context (examination of complete and closed school classes), it can also only be expanded to a limited extent. It should therefore be examined which strategies exist to enlarge the sample or which statistical approaches could be considered in the future to compensate for this weakness [e.g., Bayesian estimations (Farine and Strandburg-Peshkin, 2015)]. In addition, there are the previously described concerns about the validity of the network questions. Pilot testing could help remedy this in the future. Finally, consideration should also be given to whether certain network data (e.g., social interaction during lessons) could be collected more objectively, for example, through observations by an external person or by electronic sensors. This would help to avoid possible bias due to social desirability. A comparison of this data with qualitative interview data would offer the chance of great insights – e.g., whether the observed developments from the perspective of the teachers are due to the strong influence of individuals or tendencies of the entire group. In particular, observations would also include the opportunity to test the assumptions about the organization of the school hours (e.g., for example, the use of certain social forms in the EOtC). In a larger follow-up study, consideration should also be given to grading the networks’ interactions in terms of intensity, frequency, and reciprocity.

## 5. Conclusion

There is no doubt that both natural environments and the characteristics of EOtC concepts itself make it possible to realize new and intensified forms of social interaction. However, based on our results, but also those of previous research, it also appears that this realization and intensification of social interaction because of EOtC should not be mistakenly assumed as an automatism. On the contrary, under certain circumstances, the frequently observed and praised freedom of choice for students in EOtC or other nature-based educational and learning concepts might lead to a solidification of existing contacts and groups, as well as the social exclusion of individuals. From our perspective, in addition to the need for methodologically well-constructed research, there is also a high demand for the development and evolution of specific didactic frameworks for EOtC and other nature-based forms of teaching and learning. In countries in which EOtC historically and currently plays a larger role (especially in Scandinavia), corresponding concepts have already been adopted in ministerial recommendations. In other countries such as Germany, where the concept is still rarely used, there is a lack of such. Last but not least, the example of the PE-profile shows that objectives that go beyond academic learning–e.g., in terms of social factors–can be effectively anchored in official recommendations. Only under these conditions there seems to be a possibility that EOtC as a social situation in natural environments can unfold its full potential in terms of social interaction and thus hold a return on investment that justifies a consequent dissemination of the concept.

## Data availability statement

The original contributions presented in the study are included in the article, further inquiries can be directed to the corresponding author.

## Ethics statement

The studies involving human participants were reviewed and approved by Technical University of Munich (Institute of Medicine). Written informed consent to participate in this study was provided by the participants’ legal guardian/next of kin.

## Author contributions

The study was designed by FM and CM. Data collection was performed by JE and CM. Statistical analysis was performed by JE with the assistance of JB and CM. JE prepared the draft of the manuscript. JA, FM, JB, and CM made essential comments and substantial revisions to it. All authors contributed to the article and approved the submitted version.

## Funding

The study was conducted in cooperation with the Technical University of Munich within the programme Open Access Publishing. The authors declare that this study received funding from AOK Baden-Württemberg (public health insurance company). The funder was not involved in the study design, collection, analysis, interpretation of data, the writing of this article or the decision to submit it for publication.

## Conflict of interest

The authors declare that the research was conducted in the absence of any commercial or financial relationships that could be construed as a potential conflict of interest.

## Publisher’s note

All claims expressed in this article are solely those of the authors and do not necessarily represent those of their affiliated organizations, or those of the publisher, the editors and the reviewers. Any product that may be evaluated in this article, or claim that may be made by its manufacturer, is not guaranteed or endorsed by the publisher.

## Footnotes

^1^ https://osf.io/6unbf

## References

[ref1] Barfod K. S. (2018). Maintaining mastery but feeling professionally isolated: experienced teachers’ perceptions of teaching outside the classroom. J. Adv. Educ. Outdoor Learn. 18, 201–213. doi: 10.1080/14729679.2017.1409643

[ref2] Baron R. Byrne D. Suls J. (2000). Exploring Social Psychology. Boston: Allyn and Bacon.

[ref3] Becker C. Lauterbach G. Spengler S. Dettweiler U. Mess F. (2017). Effects of regular classes in outdoor education settings: a systematic review on students’ learning, social and health dimensions. Int. J. Environ. Res. Public Health 14:485. doi: 10.3390/ijerph14050485, PMID: 28475167 PMC5451936

[ref4] Becker C. Schmidt S. Neuberger E. Kirsch P. Simon P. Dettweiler U. (2019). Children’s cortisol and cell-free DNA trajectories in relation to sedentary behavior and physical activity in school: a pilot study. Front. Public Health 7:26. doi: 10.3389/fpubh.2019.00026, PMID: 30873396 PMC6400867

[ref5] Bentsen P. Mygind L. Elsborg B. Nielsen G. Mygind E. (2021). Education outside the classroom as upstream school health promotion: ‘adding-in’ physical activity into children’s everyday life and settings. Scand. J. Public Health 50, 303–311. doi: 10.1177/1403494821993715, PMID: 33624553

[ref6] Bentsen P. Mygind E. Randrup T. B. (2009). Towards an understanding of udeskole: education outside the classroom in a Danish context. Education 37, 29–44. doi: 10.1080/03004270802291780

[ref7] Block P. Grund T. (2014). Multidimensional homophily in friendship networks. Netw. Sci. 2, 189–212. doi: 10.1017/nws.2014.17, PMID: 25525503 PMC4267571

[ref8] Block P. Stadtfeld C. Snijders T. A. B. (2019). Forms of dependence: comparing SAOMs and ERGMs from basic principles. Sociol. Methods Res. 48, 202–239. doi: 10.1177/0049124116672680

[ref9] Bølling M. Mygind E. Mygind L. Bentsen P. Elsborg P. (2021). The association between education outside the classroom and physical activity: differences attributable to the type of space? Children 8:486. doi: 10.3390/children8060486, PMID: 34200485 PMC8227423

[ref10] Bølling M. Otte C. R. Elsborg P. Nielsen G. Bentsen P. (2018). The association between education outside the classroom and students’ school motivation: results from a one-school-year quasi-experiment. Int. J. Educ. Res. 89, 22–35. doi: 10.1016/j.ijer.2018.03.004

[ref11] Bølling M. Pfister G. U. Mygind E. Nielsen G. (2019). Education outside the classroom and pupils’ social relations? A one-year quasi-experiment. Int. J. Educ. Res. 94, 29–41. doi: 10.1016/j.ijer.2019.02.014

[ref12] Borgatti S. P. Everett M. G. Johnson J. C. (2018). Analyzing Social Networks. Thousand Oaks: Sage Publications.

[ref13] Chalkiadaki A. (2018). A systematic literature review of 21st century skills and competencies in primary education. Int. J. Instr. 11, 1–16. doi: 10.12973/iji.2018.1131a

[ref14] Deci E. Ryan R. (2000). The “what” and “why” of goal pursuits: human needs and the self-determination of behavior. Psychol. Inq. 11, 227–268. doi: 10.1207/S15327965PLI1104_01

[ref15] Dettweiler U. Lauterbach G. Becker C. Simon P. (2017). A Bayesian mixed-methods analysis of basic psychological needs satisfaction through outdoor learning and its influence on motivational behavior in science class. Front. Psychol. 8:2235. doi: 10.3389/fpsyg.2017.02235, PMID: 29312080 PMC5742242

[ref16] Doucette P. A. (2004). Walk and talk: an intervention for behaviorally challenged youths. Adolescence 39, 373–388. 15563045

[ref17] Ellinger J. Mess F. Blaschke S. Mall C. (2022a). Health-related quality of life, motivational regulation and basic psychological need satisfaction in education outside the classroom: an explorative longitudinal pilot study. BMC Public Health 22:49. doi: 10.1186/s12889-021-12450-9, PMID: 34998374 PMC8742160

[ref18] Ellinger J. von Au J. Bernhardt L. Singer M. Tangerding S. Mall C. (2022b). Eine Frage der Perspektive: die Unterrichtssituation im Draußenunterricht [A matter of perspective: the classroom situation in outdoor education]. In: Au J. von Jucker R. (eds). Draussenlernen Neue Forschungsergebnisse und Praxiseinblicke für eine Bildung für nachhaltige Entwicklung [Outdoor Learning New Research Findings and Practical Insights for Education for Sustainable Development]. Bern: hep. 387–406.

[ref19] Faber Taylor A. Wiley A. Kuo F. Sullivan W. (1998). Growing up in the inner city. Green spaces as places to grow. Environ. Behav. 30, 3–27. doi: 10.1177/0013916598301001

[ref20] Fägerstam E. (2014). High school teachers’ experience of the educational potential of outdoor teaching and learning. J. Adv. Educ. Outdoor Learn. 14, 56–81. doi: 10.1080/14729679.2013.769887

[ref21] Farine D. Strandburg-Peshkin A. (2015). Estimating uncertainty and reliability of social network data using Bayesian inference. R. Soc. Open Sci. 2:150367. doi: 10.1098/rsos.150367, PMID: 26473059 PMC4593693

[ref22] Fernandez-Rio J. Sanz N. Fernandez-Cando J. Santos L. (2017). Impact of a sustained cooperative learning intervention on student motivation. Phys. Educ. Sport Pedagog. 22, 89–105. doi: 10.1080/17408989.2015.1123238

[ref23] Fullerton C. S. Ursano R. J. (1994). Preadolescent peer friendships: a critical contribution to adult social relatedness? J. Youth Adolesc. 23, 43–63. doi: 10.1007/BF01537141

[ref24] Fyfe-Johnson A. Hazlehurst M. Perrins S. Bratman G. Thomas R. Garrett K.. (2021). Nature and children’s health: a systematic review. Pediatrics 148:e2020049155. doi: 10.1542/peds.2020-049155 34588297

[ref25] García-Carrión R. Villarejo-Carballido B. Villardón-Gallego L. (2019). Children and adolescents mental health: a systematic review of interaction-based interventions in schools and communities. Front. Psychol. 10:918. doi: 10.3389/fpsyg.2019.00918, PMID: 31068881 PMC6491840

[ref26] Gifford-Smith M. E. Brownell C. A. (2003). Childhood peer relationships: social acceptance, friendships, and peer networks. J. Sch. Psychol. 41, 235–284. doi: 10.1016/S0022-4405(03)00048-7

[ref27] Gillies R. M. Ashman A. F. (2000). The effects of cooperative learning on students with learning difficulties in the lower elementary school. J. Spec. Educ. 34, 19–27. doi: 10.1177/002246690003400102

[ref28] Glackin M. (2018). ‘Control must be maintained’: exploring teachers’ pedagogical practice outside the classroom. Br. J. Sociol. Educ. 39, 61–76. doi: 10.1080/01425692.2017.1304204

[ref29] Hanfstingl B. Andreitz I. Müller F. H. Thomas A. (2010). Are self-regulation and self-control mediators between psychological basic needs and intrinsic teacher motivation? J. Educ. Res. Online 2, 55–71. doi: 10.25656/01:4575

[ref30] Hänze M. Berger R. (2007). Cooperative learning, motivational effects, and student characteristics: an experimental study comparing cooperative learning and direct instruction in 12th grade physics classes. Learn. Instr. 17, 29–41. doi: 10.1016/j.learninstruc.2006.11.004

[ref31] Harris M. A. Orth U. (2020). The link between self-esteem and social relationships: a meta-analysis of longitudinal studies. J. Pers. Soc. Psychol. 119, 1459–1477. doi: 10.1037/pspp0000265, PMID: 31556680

[ref32] Hartmeyer R. Mygind E. (2016). A retrospective study of social relations in a Danish primary school class taught in ‘u deskole’. J. Adv. Educ. Outdoor Learn. 16, 78–89. doi: 10.1080/14729679.2015.1086659

[ref33] Hartup W. W. (1989). Social relationships and their developmental significance. Am. Psychol. 44, 120–126. doi: 10.1037/0003-066X.44.2.120

[ref34] Hartup W. W. (1999). “Peer experience and its developmental significance,” in Developmental Psychology: Achievements and Prospects. ed. Bennett M. (Hove: Psychology Press), 106–125.

[ref35] Hay D. F. Caplan M. Nash A. (2018). “The beginnings of peer relations,” in Handbook of Peer Interactions, Relationships, and Groups. eds. Bukowski W. Laursen B. Rubin K. (New York City: The Guilford Press), 200–221.

[ref36] Hills L. A. Croston A. (2012). ‘It should be better all together’: exploring strategies for ‘undoing’gender in co-educational physical education. Sport Educ. Soc. 17, 591–605. doi: 10.1080/13573322.2011.553215

[ref37] Hirsh-Pasek K. Hadani H. S. Blinkoff E. Golinkoff R. M. (2020). A new path to education form: playful learning promotes 21st century skills in school and beyond. *Policy Brookings 2020*. Available at: https://www.brookings.edu/wp-content/uploads/2020/10/Big-Ideas_Hirsh-Pasek_PlayfulLearning.pdf (Accessed August 29, 2022).

[ref38] Hunter D. R. Handcock M. S. Butts C. T. Goodreau S. M. Morris M. (2008). Ergm. A package to fit, simulate and diagnose exponential-family models for networks. J. Stat. Softw. 24:nihpa54860. doi: 10.18637/jss.v024.i03, PMID: 19756229 PMC2743438

[ref39] Hurst B. Wallace R. R. Nixon S. B. (2013). The impact of social interaction on student learning. Reading Horiz. 52, 375–398.

[ref40] Jørring A. H. Bølling M. Nielsen G. Stevenson M. P. Bentsen P. (2019). Swings and roundabouts? Pupils’ experiences of social and academic well-being in education outside the classroom. Education 48, 413–428. doi: 10.1080/03004279.2019.1614643

[ref41] Koster M. Nakken H. Pijl S. J. Van Houten E. (2009). Being part of the peer group: a literature study focusing on the social dimension of inclusion in education. Int. J. Incl. Educ. 13, 117–140. doi: 10.1080/13603110701284680

[ref42] La Guardia J. Ryan R. Couchman C. Deci E. (2000). Basic psychological needs scales. J. Pers. Soc. Psychol. 79, 367–384. PMID: 10981840 10.1037//0022-3514.79.3.367

[ref43] Lee E. Y. de Lannoy L. Li L. de Barros M. Bentsen P. Brussoni M.. (2022). Play, learn, and teach outdoors–network (PLaTO-net): terminology, taxonomy, and ontology. Int. J. Behav. Nutr. Phys. Act. 19. doi: 10.1186/s12966-022-01294-0, PMID: 35701784 PMC9199154

[ref44] Lee R. L. T. Lane S. Brown G. Leung C. Kwok S. W. H. Chan S. W. C. (2020). Systematic review of the impact of unstructured play interventions to improve young children’s physical, social, and emotional well-being. Nurs. Health Sci. 22, 184–196. doi: 10.1111/nhs.12732, PMID: 32358875

[ref45] Li J. Li J. Jia R. Wang Y. Qian S. Xu Y. (2020). Mental health problems and associated school interpersonal relationships among adolescents in China: a cross-sectional study. Child Adolesc. Psychiatry Ment. Health 14, 12–10. doi: 10.1186/s13034-020-00318-6, PMID: 32256690 PMC7106742

[ref46] Luke D. A. (2015). A User’s Guide to Network Analysis in R. Berlin: Springer.

[ref47] Ma J. (2018). Qualitative change in social situation of development as the starting point of children’s role adjustment during the transition to school. Early Child Dev. Care. 190, 750–765. doi: 10.1080/03004430.2018.1490895

[ref48] Maccoby E. E. (1988). Gender as a social category. Dev. Psychol. 24, 755–765. doi: 10.1037/0012-1649.24.6.755

[ref49] Mall C. von Au J. Dettweiler U. (2021). “Students’ appropriation of space in education outside the classroom. Some aspects on physical activity and health from a pilot study with 5th-graders in Germany,” in Nature and Health. eds. Brymer E. Rogerson M. Barton J. (London: Routledge), 223–232.

[ref50] McArdle K. Harrison T. Harrison D. (2013). Does a nurturing approach that uses an outdoor play environment build resilience in children from a challenging background? J. Adv. Educ. Outdoor Learn. 13, 238–254. doi: 10.1080/14729679.2013.776862

[ref51] McNamara L. Colley P. Franklin N. (2017). School recess, social connectedness and health: a Canadian perspective. Health Promot. Int. 32, dav102–dav402. doi: 10.1093/heapro/dav102 26497586

[ref52] Miethling W.-D. (1986). Belastungssituationen im Selbstverständnis junger Sportlehrer. Ein Beitrag zur Praxisforschung im Sportunterricht [Stressful Situations in the Self-concept of Young Physical Education Teachers. A Contribution to Practice Research in Physical Education]. Schorndorf: Verlag Karl Hofmann.

[ref53] Ministerium für Kultus, Jugend und Sport Baden-Württemberg [Ministry of Education, Youth and Sports Baden-Württemberg] (2016). Bildungsplan des Gymnasiums. Sport [Educational plan of the high school. Sports]. Available at: https://www.bildungsplaene-bw.de/site/bildungsplan/get/documents/lsbw/export-pdf/depot-pdf/ALLG/BP2016BW_ALLG_GYM_SPO.pdf (Accessed August 29, 2022).

[ref54] Moriguchi Y. (2014). The early development of executive function and its relation to social interaction: a brief review. Front. Psychol. 5:388. doi: 10.3389/fpsyg.2014.00388, PMID: 24808885 PMC4010730

[ref55] Mygind E. (2009). A comparison of childrens’ statements about social relations and teaching in the classroom and in the outdoor environment. J. Adv. Educ. Outdoor Learn. 9, 151–169. doi: 10.1080/14729670902860809

[ref56] Mygind L. Kjeldsted E. Hartmeyer R. Mygind E. Bølling M. Bentsen P. (2019). Mental, physical and social health benefits of immersive nature-experience for children and adolescents: a systematic review and quality assessment of the evidence. Health Place 58:102136. doi: 10.1016/j.healthplace.2019.05.014, PMID: 31220797

[ref57] Nynke M. D. Niezink T. Snijders M. van Duijn M. (2019). No longer discrete: modeling the dynamics of social networks and continuous behavior. Sociol. Methodol. 49, 295–340. doi: 10.1177/0081175019842263

[ref58] Pachucki M. C. Ozer E. J. Barrat A. Cattuto C. (2015). Mental health and social networks in early adolescence: a dynamic study of objectively-measured social interaction behaviors. Soc. Sci. Med. 125, 40–50. doi: 10.1016/j.socscimed.2014.04.015, PMID: 24797692

[ref59] Perry D. G. Pauletti R. E. Cooper P. J. (2019). Gender identity in childhood: a review of the literature. Int. J. Behav. Dev. 43, 289–304. doi: 10.1177/0165025418811129

[ref60] Powlishta K. K. (2004). “Gender as a social category: intergroup processes and gender-role development,” in The Development of the Social Self. eds. Bennett M. Sani F. (London: Psychology Press), 103–133.

[ref61] Putra I. Astell-Burt T. Cliff D. P. Vella S. A. John E. E. Feng X. (2020). The relationship between green space and prosocial behaviour among children and adolescents: a systematic review. Front. Psychol. 11:859. doi: 10.3389/fpsyg.2020.00859, PMID: 32425867 PMC7203527

[ref62] Ridgeway C. Smith-Lovin L. (1999). The gender system and interaction. Annu. Rev. Sociol. 25, 191–216. doi: 10.1146/annurev.soc.25.1.191

[ref63] Ripley R. M. Snijders T. A. B. Boda Z. Vörös A. Preciado P. (2012). Manual for Siena version 4.0. Available at: https://www.stats.ox.ac.uk/~snijders/siena/s_man401.pdf (Accessed August 29, 2022).

[ref64] Roberts A. Hinds J. Camic P. M. (2020). Nature activities and well-being in children and young people: a systematic literature review. J. Adv. Educ. Outdoor Learn. 20, 298–318. doi: 10.1080/14729679.2019.1660195

[ref65] Roe J. Aspinall P. (2011). The emotional affordances of forest settings: an investigation in boys with extreme behavioural problems. Landsc. Res. 36, 535–552. doi: 10.1080/01426397.2010.543670

[ref66] Roseth C. J. Johnson D. W. Johnson R. T. (2008). Promoting early adolescents' achievement and peer relationships: the effects of cooperative, competitive, and individualistic goal structures. Psychol. Bull. 134, 223–246. doi: 10.1037/0033-2909.134.2.223, PMID: 18298270

[ref67] Ryan R. M. Deci E. L. (2017). Self-Determination Theory: Basic Psychological Needs in Motivation, Development, and Wellness. New York: Guilford Publications.

[ref68] Sarason S. B. Klaber M. (1985). The school as a social situation. Annu. Rev. Psychol. 36, 115–140. doi: 10.1146/annurev.ps.36.020185.000555 20809781

[ref69] Schneller M. B. Duncan S. Schipperijn J. Nielsen G. Mygind E. Bentsen P. (2017). Are children participating in a quasi-experimental education outside the classroom intervention more physically active? BMC Public Health 17:523. doi: 10.1186/s12889-017-4430-5, PMID: 28549469 PMC5446688

[ref70] Seppala E. Rossomando T. Doty J. R. (2013). Social connection and compassion: important predictors of health and well-being. Soc. Res. 80, 411–430. doi: 10.1353/sor.2013.0027

[ref71] Shrum W. Cheek N. H. M. D. Jr. Hunter S. (1988). Friendship in school: gender and racial homophily. Sociol. Educ. 61, 227–239. doi: 10.2307/2112441

[ref72] Shumate M. Palazzolo E. T. (2010). Exponential random graph (p*) models as a method for social network analysis in communication research. Commun. Methods Meas. 4, 341–371. doi: 10.1080/19312458.2010.527869

[ref73] Simmel G. (2013). “Soziologie: Untersuchungen über die Formen der Vergesellschaftung [Sociology: studies on the forms of socialisation],” in Hauptwerke der Emotionssoziologie [Major Works in the Sociology of Emotion]. eds. Senge K. Schützeichel R. (Wiesbaden: Springer), 311–332.

[ref74] Snijders T. van de Bunt G. Steglich C. (2010). Introduction to stochastic actor-based models for network dynamics. Soc. Networks 32, 44–60. doi: 10.1016/j.socnet.2009.02.004

[ref75] Sugiyama T. Leslie E. Giles-Corti B. Owen N. (2008). Associations of neighbourhood greenness with physical and mental health: do walking, social coherence and local social interaction explain the relationships? J. Epidemiol. Community Health 62:e9. doi: 10.1136/jech.2007.064287 18431834

[ref76] Torkos H. (2017). Social and psychological aspects of outdoor education. Agora Psycho Pragmat. 11, 215–223.

[ref77] Tremblay M. S. Gray C. Babcock S. Barnes J. Bradstreet C. C. Carr D.. (2015). Position statement on active outdoor play. Int. J. Environ. Res. Public Health 12, 6475–6505. doi: 10.3390/ijerph120606475, PMID: 26062040 PMC4483712

[ref78] van Laar E. van Deursen A. J. van Dijk J. A. de Haan J. (2020). Determinants of 21st-century skills and 21st-century digital skills for workers: a systematic literature review. SAGE Open 10:215824401990017. doi: 10.1177/2158244019900176

[ref79] Van Ryzin M. J. Roseth C. J. (2018). Cooperative learning in middle school: a means to improve peer relations and reduce victimization, bullying, and related outcomes. J. Educ. Psychol. 110, 1192–1201. doi: 10.1037/edu0000265, PMID: 30911200 PMC6430212

[ref80] Waite S. Bølling M. Bentsen P. (2016). Comparing apples and pears?: a conceptual framework for understanding forms of outdoor learning through comparison of English Forest schools and Danish udeskole. Environ. Educ. Res. 22, 868–892. doi: 10.1080/13504622.2015.1075193

[ref81] Wang Y. Tian L. Huebner S. (2019). Basic psychological needs satisfaction at school, behavioral school engagement, and academic achievement: longitudinal reciprocal relations among elementary school students. Contemp. Educ. Psychol. 56, 130–139. doi: 10.1016/j.cedpsych.2019.01.003

